# Supplementary material for: β-Xylosidase SRBX1 Activity from Sporisorium reilianum and Its Synergism with Xylanase SRXL1 in Xylose Release from Corn Hemicellulose
Source: J Fungi (Basel). 2022 Dec 13;8(12):1295. doi: 10.3390/jof8121295 (PMC9781407; doi:10.3390/jof8121295)
Supplement: Supplementary file 1 [file jof-08-01295-s001.zip › jof-2047201-supplementary.pdf]

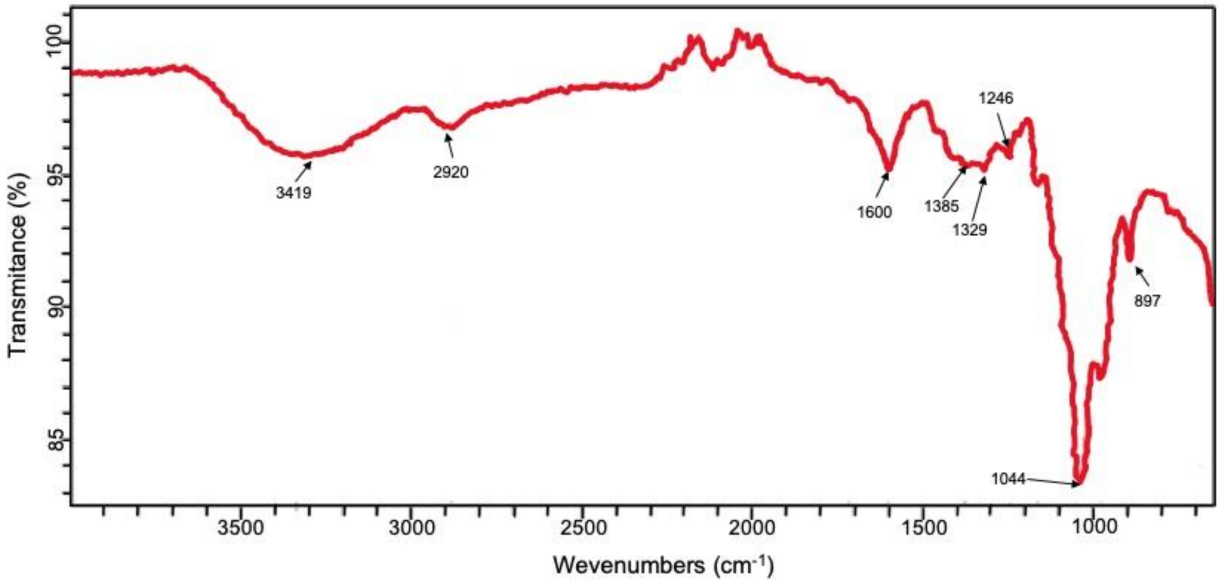

Supplementary Figure S1. FT-IR spectra of the hemicellulose obtained from corn cobs. The arrows indicate the characteristic wavenumbers of this polymer.

1 MKAAVKSLLVLSIYAANVLSAQAASLEHLNMLFHTRQADASNSTDSSSVN 50
51 WNISSSDNSALATAPLAGTFYRGGPALADANSPFHPVTGNGGWEWAVVKA 100
101 RSVVGQLTLEEKVNLTAGITGGRCEGTLGRVDRFGIPELCFQDGPAGFRA 150
151 SDFVTVFPAGVTTAATWNRDLIYKRAAALGAEFVAKGVNVHLGPVTGGPL 200
201 GRSPFQGRNWEGFGPDPYLHGEAAYYTVSGTQSAGVISTAKHFLAYEQET 250
251 YRQLYAASDPFTLNPNNNTELTYSANLDDRTLHELYLWPFMNAVRAGTGA 300
301 LVCVYNRVNSTQGCESSKLLNE**ILKDELDYQGFVVTDWSA**AFNTSNTYNG 350
351 GSDVVMPGGMTGGYKNLVGGSDLVRALNAGEVKIERINDGITRLLTQWYL 400
401 RGQDKGYPTVSYKDGYQNTIFNGTVVNEHRDVQGDHRKIVKEIGEEAVTL 450
451 IYNKRSNKAGPQGNTDFGLGLPLAKKARVAVFGSDAGPNPYGANACQDWI 500
501 GLGSQLCPANATSNGTQAVGWGSGAGFFPYLIDPLAGISEVAKENRGAVL 550
551 HNLNDVGDDKNQMYVKQQAGLADASLVFVQARSGEDSDRHSLRLDADGDE 600
601 LIKLVASQSNNTIVVMHTVGPVLMGDWFDHPNITALVLPHLPGQESGSSL 650
651 ARVLYGDVNPSGKMPYSMLSDEDAKRYPKIVGSPASDPQVDFYDGLYIDY 700
701 RAWDKMGLKPLIPFGHGISYTNYSYSNLHIQKAGDNCYAPSAFSGSFKSD 750
751 KQPGGPGSLFQYLVEVSADVQNVGAMAGDEVAQLYVGYPEAANAPIKQLR 800
801 GFDKVQGLEPGAASKQATFKLAKRDFSVWDVVKQKFEVVDGEYKIWVGKS 850
851 SRMSDLTLKGSVTMQNGMVVGMSS 874

Supplementary Figure S2. Predicted theoretical amino acid sequence of β-xylosidase SRBX1 from *S. reilianum*. The signal peptide is underlined, and an arrow indicates the cleavage site. The motive of the glycosyl hydrolases family 3 is indicated in bold letters. The glycosylation sites are highlighted in black. The sequences of the peptides obtained from the sequencing are highlighted in gray.
